# Associations of serum high-sensitivity C-reactive protein and prealbumin with coronary vessels stenosis determined by coronary angiography and heart failure in patients with myocardial infarction

**DOI:** 10.5937/jomb0-37847

**Published:** 2023-01-20

**Authors:** Yun Zhu, Zhen Yu, Ronggui Xu, Beibei Wang, Yiqun Lou, Na Zhang, Ziyin Chen

**Affiliations:** 1 Xianju County Peoples Hospital, Department of Cardiovascular Medicine, Taizhou, China; 2 Jinan Central Hospital, Department of Centralized Dispensing of Intravenous Drugs, Jinan, China; 3 Xianju County Peoples Hospital, China, Department of Cardiovascular Medicine, Taizhou

**Keywords:** high-sensitivity C-reactive protein, prealbumin, myocardial infarction, coronary artery disease, visokoosetljivi C-reaktivni protein, prealbumin, infarkt miokarda, koronarna bolest

## Abstract

**Background:**

To explore the associations of serum high-sensitivity C-reactive protein (hs-CRP) and prealbumin (PAB) with the number of diseased coronary vessels, degree of stenosis and heart failure in patients with myocardial infarction (MI).

**Methods:**

A total of 39 MI patients treated in the Cardiology were selected as the observation group, and another 41 patients with normal results of coronary angiography during the same period were selected as the control group. The general data of patients were recorded in detail, the content of serum hs-CRP and PAB in the peripheral blood was detected, and the number of diseased coronary vessels and the degree of stenosis were detected via coronary angiography.

**Results:**

Compared with those in control group, the blood pressure and heart rate significantly rose, the content of indexes related to the severity of MI were significantly increased, the content of hs-CRP was significantly increased, and the content of PAB was significantly decreased in observation group. Hs-CRP was positively correlated with the number of diseased coronary vessels, degree of stenosis and heart failure in patients, but PAB was negatively correlated with the above factors. The survival rate of MI patients with high content of hs-CRP was obviously lower than that of patients with low content of hsCRP

**Conclusions:**

Serum hs-CRP and PAB are closely associated with the number of diseased coronary vessels, degree of stenosis and heart failure in MI patients.

## Abstract (sr)

**Uvod:**

Cilj je bio da se istraži povezanost serumskog visokoosetljivog C-reaktivnog proteina (hs-CRP) i prealbumina (PAB) sa brojem obolelih koronarnih sudova, stepenom stenoze i srčanom insuficijencijom kod pacijenata sa infarktom miokarda (MI).

**Metode:**

Za posmatračku grupu izabrano je ukupno 39 pacijenata sa IM lečenim kardiološki, a za kontrolnu grupu je izabran još 41 pacijent sa normalnim rezultatima koronarne angiografije u istom periodu. Detaljno su evidentirani opšti podaci pacijenata, detektovan je sadržaj serumskog hs-CRP i PAB u perifernoj krvi, a koronarografijom je utvrđen broj obolelih koronarnih sudova i stepen stenoze.

**Rezultati:**

U poređenju sa onima u kontrolnoj grupi, znacajno su porasli krvni pritisak i puls, znacajno je povećan sadržaj indeksa koji se odnose na težinu IM, znacajno povećan sadržaj hs-CRP, a znacajno smanjen sadržaj PAB u posmatrackoj grupi. hs-CRP je bio u pozitivnoj korelaciji sa brojem obolelih koronarnih sudova, stepenom stenoze i srčanom insuficijencijom kod pacijenata, ali je PAB bio u negativnoj korelaciji sa navedenim faktorima. Stopa preživljavanja pacijenata sa IM sa visokim sadržajem hs-CRP bila je očigledno niža nego kod pacijenata sa niskim sadržajem hs-CRP.

**Zaključak:**

Serumski hs-CRP i PAB su usko povezani sa brojem obolelih koronarnih sudova, stepenom stenoze i srčanom insuficijencijom kod pacijenata sa IM.

## Introduction

Acute myocardial infarction (AMI) is a heart disease, whose pathogenesis is that persistent myocardial ischemia and hypoxia are caused by interruption or decline in coronary blood flow due to various factors based on the coronary artery disease, ultimately leading to myocardial cell necrosis, usually accompanied by such clinical complications as cardiac insufficiency and heart failure [1] [2]. With the intensification of population aging in China in recent years, the morbidity rate of AMI has increased year by year, making AMI one of the common diseases causing death and affecting the quality of life [3]. It has been a major concern for clinicians and researchers to judge the number of diseased coronary vessels, degree of stenosis and incidence of heart failure through clinical indexes and realize early prevention and treatment. High-sensitivity C-reactive protein (hs-CRP) is a sensitive marker for inflammation and atherosclerosis in the body. As a cytokine, it is involved in the formation of vascular plaques and the aggregation and adhesion of leukocytes, and it is closely related to the vascular endothelial injury [4] and the occurrence and development of AMI [5]. Prealbumin (PAB) synthesized and released by hepatocytes is an acute phase reactive protein, which can effectively reduce the damage of toxic metabolites to the body, and has a close correlation with the severity of AMI [6]. Sun et al. [7] found that PAB can be significantly consumed in the case of heart failure, so that the content of PAB in the peripheral blood of patients greatly declines. In this study, the associations of serum hs-CRP and PAB with the number of diseased coronary vessels, degree of stenosis and heart failure in AMI patients were analyzed, so as to provide a theoretical basis for the early diagnosis and treatment of AMI patients in clinic.

## Materials and methods

### Objects of study

AMI patients treated in our hospital were selected, and they met the diagnostic criteria for AMI of the American College of Cardiology (ACC), the European Society of Cardiology (ESC) and the American Heart Association (AHA): >50% stenosis of more than 1 vessel definitely confirmed by coronary angiography. All patients enrolled underwent echocardiography and coronary angiography to confirm the diagnosis. Exclusion criteria: 1) Patients with chronic wasting diseases, such as malignant tumors, 2) those with severe dysfunction of liver or kidney, 3) those with acute infections recently or hematological diseases, 4) those with autoimmune diseases, or 5) those with a history of myocardial diseases. In observation group (n=39), there were 21 males and 18 females aged 48-82 years old. Another 41 patients without coronary artery disease according to coronary angiography during the same period were selected as the control group, including 20 males and 21 females aged 46-79 years old. The patients in both groups signed the informed consent and agreed to be enrolled in the study. The experimental scheme in this study was approved by the Ethics Committee of Xianju County Peoples Hospital.

### Research methods

### General data of patients

The general data of patients were recorded in detail, including age, gender, height, weight, blood pressure and heart rate at admission, smoking history, medical history and medication history.

### Judgment of severity of coronary artery disease

Coronary angiography was performed for all patients by the same physician in the Cardiovascular Intervention Department using the Judkin's method. Each blood vessel was subjected to projection at more than 3 positions, and the severity of disease and the number of diseased coronary vessels were evaluated independently. According to the angiography results, over 50% stenosis indicated the positive. The eight main vascular segments selected included the proximal right coronary artery, the middle right coronary artery, the proximal circumflex branch, the middle circumflex branch, the first diagonal branch, the middle anterior descending branch, the proximal anterior descending branch and the left main coronary artery. The coronary artery diseases were classified into single-vessel disease, double-vessel disease and multivessel disease.

### Score of coronary artery stenosis

The degree of coronary artery stenosis was evaluated using the Gensini scoring system according to the criteria of ACC <25% stenosis: 0 points, 25-49% stenosis: 1 point, 50-74% stenosis: 2 points, 75-99% stenosis: 3 points, and 100% stenosis: 4 points. The score of disease was processed as follows: left main coronary artery disease x 5, proximal circumflex branch disease and proximal anterior descending branch disease x 2.5, middle anterior descending branch disease x 1.5, and diseases at other sites x 1. Finally, the score of coronary artery stenosis was the sum of scores of the 8 vessels.

### Laboratory examination

The venous blood was drawn immediately after admission, placed for 30 min and centrifuged at 3,000 rpm for 15 min. The supernatant was harvested and stored in an ultra-low temperature refrigerator for later use. The content of serum hs-CRP and PAB was detected by professional technicians using an automatic electrochemiluminescence immunoassay analyzer (E601, Roche, USA) and a full-automatic biochemical analyzer, respectively, strictly in accordance with the instructions.

The venous blood was drawn immediately after admission, placed for 30 min and centrifuged at 3,000 rpm for 15 min. Then the content of AMI-related indexes was detected, including myoglobin (MYO), creatine kinase-MB (CK-MB), N-terminal probrain natriuretic peptide (NT-proBNP) and cardiac troponin I (cTnI).

### Follow-up

The patients were followed up during hospitalization and via telephone for 2 years. The incidence of heart failure was recorded, and the patients with heart failure were subjected to Killip cardiac function grading (grade I-IV). Based on the content of hs-CRP, the patients in observation group were divided into high-concentration group and low-concentration group. The survival time of patients was recorded, and the survival curves were plotted. The detailed records about follow-up, examinations and hospitalization were kept.

### Statistical analysis

The data in this study were expressed as mean ± standard deviation. Statistical Product and Service Solutions (SPSS) 22.0 software (SPSS Inc., Chicago, IL, USA) was used for the data analysis. χ^2^ test was performed for the intergroup analysis of enumeration data, and one-way analysis of variance was used for the comparison among groups. Homogeneity test of variance was performed. Bonferroni's method was adopted for pairwise comparison in the case of homogeneity of variance, while Welch's method was adopted in the case of heterogeneity of variance. The survival status of patients was evaluated using Kaplan-Meier analysis, and the associations of indexes were detected using Pearson correlation analysis. P<0.05 suggested that the difference was statistically significant.

## Results

### General data of patients

The general data of patients in the two groups were recorded in detail after enrollment. As shown in Table 1, there were no statistically significant differences in age, gender, body mass index (BMI) and smoking history between observation group and control group (P>0.05). The blood pressure and heart rate in observation group were significantly higher than those in control group (P<0.05).

**Table 1:** General data of patients.

Item	Observation group (n=39)	Control group (n=41)	P
Age (Y)	68.16±12.76	66.33±11.82	0.076
Gender (male/female)	(21/18)	(20/21)	
BMI (kg/m^2^)	23.06±2.93	22.87±2.65	0.081
Blood pressure(mmHg)	106.32±13.25/163.78±6.23	96.17±10.26/143.17±17.23	0.008
Heart rate (beats/min)	82.23±12.56	71.67±10.52	0.047
Smoking (n, %)	(16, 41.03%)	(15, 36.58%)	0.069

### Content of indexes related to severity of MI

The fasting peripheral blood was drawn in the two groups to detect the content of MI-related indexes. As shown in Table 2, the content of MYO, CK-MB, NT-proBNP and cTnI in the peripheral blood significantly rose in observation group compared with those in control group (P<0.01).

**Table 2:** Content of indexes related to severity of MI.

Index	Observation group (n=39)	Control group (n=41)	P
MYO (IU/L)	276.53±176.21	36.85±12.67	<0.001
CK-MB (IU/L)	85.16±39.22	0.38±2.07	<0.001
NT-proBNP (pg/mL)	874.25±257.32	138.77±42.63	<0.001
cTnI (IU/L)	22.56±16.72	0.12±0.36	<0.001

### Content of serum hs-CRP and PAB

The fasting peripheral blood was drawn in the two groups to detect the content of serum hs-CRP and PAB. The results showed that observation group had obviously increased content of hs-CRP (P<0.01), but obviously decreased content of PAB in the peripheral blood compared with control group (P<0.01) (Table 3).

**Table 3:** Content of serum hs-CRP and PAB.

Index	Observation group (n=39)	Control group (n=41)	P
hs-CRP (mg/L)	15.87±6.12	2.63±3.37	<0.001
PAB (mg/L)	139.36±40.29	305.23±23.56	<0.001

### Analysis of correlations of serum hs-CRP with number of diseased coronary vessels, degree of stenosis and heart failure

**Figure 1:**
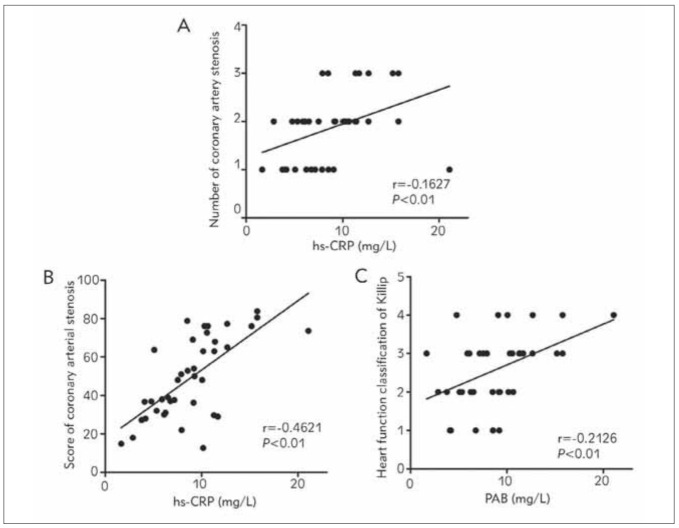
Analysis of correlations of serum hs-CRP with number of diseased coronary vessels, degree of stenosis and heart failure. (A) Correlation of serum hs-CRP with number of diseased coronary vessels. (B) Correlation of serum hs-CRP with degreeof stenosis. (C) Correlation of serum hs-CRP with heart failure. The content of serum hs-CRP in AMI patients was positivelycorrelated with the number of diseased coronary vessels, degree of stenosis and heart failure (P<0.01).

According to the Pearson correlation analysis (Figure 1), the content of hs-CRP in the peripheral blood of AMI patients was positively correlated with the number of diseased coronary vessels (r=0.1627, P<0.01), degree of stenosis (r=0.4621, P<0.01) and heart failure (r=0.2126, P<0.01).

### Analysis of correlations of PAB with number of diseased coronary vessels, degree of stenosis and heart failure

**Figure 2:**
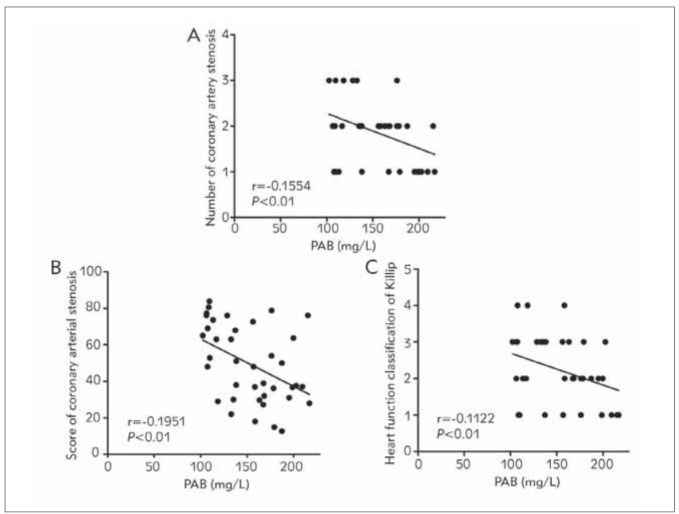
Analysis of correlations of PAB with number of diseased coronary vessels, degree of stenosis and heart failure. (A)Correlation of PAB with number of diseased coronary vessels. (B) Correlation of PAB with degree of stenosis. (C) Correlationof PAB with heart failure. The content of PAB in AMI patients was negatively correlated with the number of diseased coronaryvessels, degree of stenosis and heart failure (P<0.01).

According to the Pearson correlation analysis (Figure 2), the content of PAB in the peripheral blood of AMI patients was negatively correlated with the number of diseased coronary vessels (r=-0.1554, P<0.01), degree of stenosis (r=-0.1951, P<0.01) and heart failure (r=-0.1122, P<0.01).

### Survival analysis

**Figure 3:**
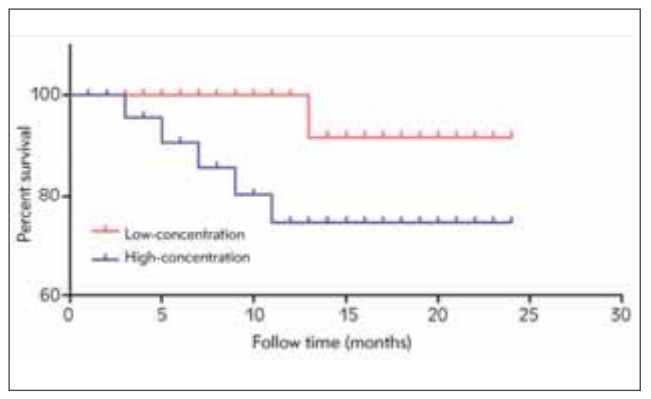
Survival analysis of MI patients. The survival ratewas far lower in high-concentration group than that in low-concentration group (P<0.05).

Based on the content of hs-CRP in the peripheral blood, the patients in observation group were divided into high-concentration group and low-concentration group. These patients were followed up for 2 years, and the survival curves were plotted. As shown in Figure 3, the survival rate was far lower in high-concentration group than that in low-concentration group (P<0.05).

## Discussion

The pathogenesis of AMI is that secondary thrombosis occurs due to coronary artery erosion or unstable plaque rupture, further causing coronary artery occlusion and myocardial ischemic necrosis [8]. hs-CRP is a classic inflammatory marker, and a reactive protein secreted by the liver upon the stimulation of inflammatory factors such as IL-6 and IL-1, which can effectively reflect the degree of coronary sclerosis and the inflammatory process in the body. In clinic, hs-CRP and myocardial injury markers are jointly used to diagnose AMI [9] [10]. Ljuca et al. [11] found that cardiovascular events such as ischemic cardiomyopathy and AMI can significantly raise the content of hs-CRP in the peripheral blood of patients. Hs-CRP can be used to effectively assess the inflammatory response in vivo and the severity of AMI, and it is closely related to the morbidity and mortality rates of cardiovascular events [12]. Besides, PAB is an acute phase reactive protein able to not only effectively evaluate the synthetic ability of the liver, but also reflect the severity of inflammatory response in patients with cardiovascular disease [13]. Matsunaga et al. [14] found that the content of PAB in the peripheral blood of patients with unstable angina pectoris is far lower than that in normal people. In this study, the content of hs-CRP and PAB in the peripheral blood of MI patients was detected. The results manifested that the content of hs-CRP in the peripheral blood of MI patients was obviously higher than that in normal people, while the content of PAB showed the opposite result. The content of hs-CRP was closely related to the severity of MI, and its increase in the peripheral blood of MI patients can significantly raise the mortality rate of patients. Fu et al. [15] showed that the elevation of hs-CRP will increase the incidence rate of adverse cardiovascular events, and cause vascular stenosis or stent thrombosis, and even death of patients.

In a large-scale retrospective study of Kalyoncuoglu et al. [16] on patients who died of cardiovascular events, it was found that hs-CRP is closely related to the mortality rate of patients with cardiovascular disease. Chi et al. [17] argued that hs-CRP has a close correlation with the degree of inflammatory response in the MI region. In this study, the correlations of the content of serum hs-CRP with the number of diseased coronary vessels, degree of stenosis and heart failure in MI patients were analyzed. The results manifested that hs-CRP was positively correlated with the number of diseased coronary vessels, degree of stenosis and heart failure. High-concentration hs-CRP can lead to vascular intima injury, vasoconstriction and unstable plaque shedding, ultimately accelerating the occurrence and development of MI [18]. Shimizu et al. [19] studied and found that hs-CPR is one of the best markers predicting MI currently. Furthermore, this study confirmed that the content of PAB in the peripheral blood of MI patients was negatively correlated with the number of diseased coronary vessels, degree of stenosis and heart failure. Wang et al. [20] found that the content of PAB in the peripheral blood is of important significance for predicting the occurrence and development of adverse events in MI patients, and PAB can serve as an independent predictive factor for MI complicated with heart failure. Moreover, Wang et al. [21] found that the content of PAB can reflect the area of MI to a certain extent.

## Conclusions

In conclusion, hs-CRP and PAB in the peripheral blood of MI patients are closely correlated with the number of diseased coronary vessels, degree of stenosis and heart failure, so they can be used as predictors of MI, which provide a theoretical basis for the accurate clinical diagnosis of MI.

## Dodatak

### Conflict of interest statement

All the authors declare that they have no conflict of interest in this work.

## References

[1] Bhuller S B, Hasan S R, Weaver J, Lieser M (2019). Acute myocardial infarction following penetrating thoracic trauma: A case report and review of literature. Int J Surg Case Rep.

[2] Khowaja S, Karim M, Zahid M, Zahid A, Ahmed S, Kazmi K, et al (2019). Impact of temperature variation on acute myocardial infarction in Karachi, Pakistan. Cureus.

[3] Ortega-Rodríguez A C, Marín-Jáuregui L S, Martínez-Shio E, Hernández C B, González-Amaro R, Escobedo-Uribe C D, et al (2020). Altered NK cell receptor repertoire and function of natural killer cells in patients with acute myocardial infarction: A three-month follow-up study. Immunobiology.

[4] Ridker P M, MacFadyen J G, Glynn R J, Bradwin G, Hasan A A, Rifai N (2020). Comparison of interleukin-6, C-reactive protein, and low-density lipoprotein cholesterol as biomarkers of residual risk in contemporary practice: secondary analyses from the Cardiovascular Inflammation Reduction Trial. Eur Heart J.

[5] Sara Cetin S, Nar R, Kilic O, Mehmet Furkan O, Gunver G, Cihan Ilyas S, 5. Gokay Nar (2022). Is serum fibroblast growth factor 21 associated with the severity or presence of coronary artery disease?. J Med Biochem.

[6] Dag Z, Koseoglu H, Kekilli M (2020). The use of prealbumin as a predictor of malnutrition in cirrhotic patients and the effect of nutritional support in patients with low prealbumin levels. Turk J Med Sci.

[7] Sun D W, An L, Lv G Y (2020). Albumin-fibrinogen ratio and fibrinogen-prealbumin ratio as promising prognostic markers for cancers: An updated meta-analysis. World J Surg Oncol.

[8] Sun Z, Pang S, Cui Y, Yan B (2019). Genetic and functional variants analysis of the GATA6 gene promoter in acute myocardial infarction. Front Genet.

[9] Nikorowitsch J, Borchardt T, Appelbaum S, Ojeda F, Lackner K J, Schnabel R B, et al (2020). Cardio-renal biomarker soluble urokinase-type plasminogen activator receptor is associated with cardiovascular death and myocardial infarction in patients with coronary artery disease independent of troponin, C-reactive protein, and renal function. J Am Heart Assoc.

[10] Cosentino N, Genovese S, Campodonico J, Bonomi A, Lucci C, Milazzo V, et al (2019). High-sensitivity C-reactive protein and acute kidney injury in patients with acute myocardial infarction: A prospective observational study. J Clin Med.

[11] Ljuca F, Hadžiefendić B, Jahić E, Tihić N, Lukić S (2019). Pentraxin 3 might be better prognostic serum marker than IL-6, IL-10, and high-sensitivity C-reactive protein for major adverse cardiovascular events in patients with ST-elevation myocardial infarction after bare-metal stent implantation. Saudi Med J.

[12] Foroughinia F, Tabibi A A, Javanmardi H, Safari A, Borhani-Haghighi A (2019). Association between high sensitivity C-reactive protein (hs-CRP) levels and the risk of major adverse cardiovascular events (MACE) and/or microembolic signals after carotid angioplasty and stenting. Caspian J Intern Med.

[13] Hong Y, Seese L, Hickey G, Mathier M, Thoma F, Kilic A (2020). Preoperative prealbumin does not impact outcomes after left ventricular assist device implantation. J Card Surg.

[14] Matsunaga T, Miyata H, Sugimura K, Motoori M, Asukai K, Yanagimoto Y, et al (2020). Prognostic significance of C-reactive protein-to-prealbumin ratio in patients with esophageal cancer. Yonago Acta Med.

[15] Fu E L, Franko M A, Obergfell A, Dekker F W, Gabrielsen A, Jernberg T, et al (2019). High-sensitivity C-reactive protein and the risk of chronic kidney disease progression or acute kidney injury in post-myocardial infarction patients. Am Heart J.

[16] Kalyoncuoglu M, Durmus G (2020). Relationship between C-reactive protein-to-albumin ratio and the extent of coronary artery disease in patients with non-ST-elevated myocardial infarction. Coron Artery Dis.

[17] Al A Z, Habib S S, Marzouk A (2019). Predictive value of high sensitivity C-reactive protein on progression to heart failure occurring after the first myocardial infarction. Vasc Health Risk Manag.

[18] Liu Y, Jia S D, Yao Y, Tang X F, Xu N, Jiang L, et al (2020). Impact of high-sensitivity C-reactive protein on coronary artery disease severity and outcomes in patients undergoing percutaneous coronary intervention. J Cardiol.

[19] Shimizu T, Suwa S, Dohi T, Wada H, Miyauchi K, Shitara J, et al (2019). Clinical significance of high-sensitivity C-reactive protein in patients with preserved renal function following percutaneous coronary intervention. Int Heart J.

[20] Wang J, Xi H, Zhang K, Li Z, Li L, Chen J, et al (2020). Circulating C-reactive protein to prealbumin ratio and prealbumin to fibrinogen ratio are two promising inflammatory markers associated with disease activity in rheumatoid arthritis. Clin Lab.

[21] Wang W, Wang C S, Ren D, Li T, Yao H C, Ma S J (2018). Low serum prealbumin levels on admission can independently predict in-hospital adverse cardiac events in patients with acute coronary syndrome. Medicine (Baltimore).

